# 冷凝收集-离子色谱法测定呼出气中的有机酸和阴离子

**DOI:** 10.3724/SP.J.1123.2023.07016

**Published:** 2024-03-08

**Authors:** Lu GAN, Yangye ZHOU, Qinqin FANG, Jianjun XU, Suqing CHEN, Yan ZHU, Chengzhu NI

**Affiliations:** 1.浙江大学校医院, 浙江 杭州 310027; 1. Zhejiang University Hospital, Hangzhou 310027, China; 2.浙江大学化学系, 浙江 杭州 310028; 2. Department of Chemistry, Zhejiang University, Hangzhou 310028, China; 3.浙江骥翔新材料有限公司, 浙江 台州 317300; 3. Zhejiang Jixiang New Material Co. Ltd., Taizhou 317300, China; 4.台州学院医药化工学院, 浙江 台州 318000; 4. School of Pharmaceutical and Chemical Engineering, Taizhou University, Taizhou 318000, China; 5.浙江省微量有毒化学物健康风险评估技术研究重点实验室, 浙江 杭州 310028; 5. Key Laboratory of Health Risk Appraisal for Trace Toxic Chemicals of Zhejiang Province, Hangzhou 310028, China; 6.台州市疾病预防控制中心, 浙江 台州 318000; 6. Taizhou Center for Disease Control and Prevention, Taizhou 318000, China

**Keywords:** 离子色谱, 有机酸, 阴离子, 呼出气冷凝液, ion chromatography (IC), organic acids, anions, exhaled breath condensation (EBC)

## Abstract

建立了一种非侵入式冷凝收集-离子色谱方法测定人体呼出气中乳酸、甲酸、乙酸、丙酮酸、Cl^-^、NO_2_^-^、NO_3_^-^、SO_4_^2-^。搭建自制呼出气冷凝装置,该装置包括吹气口、与吹气口相连的单向阀和流量计、置于半导体冷凝装置中的冷阱以及一次性冷凝收集管。通过呼出气冷凝装置对人体呼出气进行收集,利用离子色谱对冷凝液(EBC)中有机酸和阴离子的含量进行检测。优化采集冷阱温度和采集流量,得到冷阱最佳冷凝温度为-15 ℃,呼气流量为15 L/min。采用1.5 mmol/L碳酸钠和3 mmol/L碳酸氢钠混合溶液作为流动相,泵流速为0.8 mL/min,分析柱为IC-SA3 (250 mm×4.0 mm),柱温为45 ℃。8种有机酸和阴离子的线性范围均为0.1~10.0 mg/L,相关系数均≥ 0.999 3。在进样量为100 μL时,方法的检出限为0.0017~0.0150 mg/L(S/N=3),定量限为0.0057~0.0500 mg/L(S/N=10)。方法的日内和日间精密度均≤ 7.50%(n=5)。采用该方法对5位健康受试者呼出气中的有机酸和阴离子进行检测,得到8种有机酸和阴离子的含量为0.18~42.3 ng/L。在10 km的长跑运动过程中,除了一位受试者代谢异常,其余受试者呼出气中的有机酸和阴离子含量总体变化趋势为先增加后减小。本方法采样过程简单,精密度好,且没有副作用,受试者不会产生任何明显不适或风险,可为日后人体代谢物的研究提供实验思路和理论依据。

## Abstract (en)

A non-invasive condensation collection-ion chromatography method was established for the determination of organic acids and anions including lactic acid, formic acid, acetic acid, pyruvic acid, chloride, nitrate, nitrite, and sulfate in the exhaled breath of humans. The breath exhaled was condensed and collected using a home-made exhaled breath condensation equipment. This equipment included a disposable mouthpiece as a blow-off port, one-way valve and flow meter, cold trap, disposable condensate collection tube placed in the cold trap, and gas outlet. A standard sampling procedure was used. Before collection, the collection temperature and sampling volume were set on the instrument control panel, and sampling was started when the cold-trap temperature dropped to the set value, while maintaining the balance. Subjects were required to gargle with pure water before sampling. During the sampling process, the subjects were required to inhale deeply until the lungs were full of gas and then exhale evenly through the air outlet. When the set volume was collected, the instrument made a prompt sound; then, the collection was immediately ended, the expiration time was recorded, and the average collection flow was calculated according to the expiration time and sampling volume. After collection, the disposable condensation collection tube was immediately taken out, sealed, and stored in the refrigerator at -20 ℃ away from light, and immediately used for further testing. The organic acids and anions in exhaled breath condensation (EBC) were filtered through a 0.22 μm membrane filter before injection and detected by ion chromatography with conductivity detection. Factors such as collection temperature and collection flow rate during condensation collection were optimized. The optimal cooling temperature was set at -15 ℃, and the optimal exhaled breath flow rate was set at 15 L/min. The mobile phase consisted of a mixture of sodium carbonate (1.5 mmol/L) and sodium bicarbonate (3 mmol/L). The flow rate was 0.8 mL/min, and the injection volume was 100 μL. An IC-SA3 column (250 mm×4.0 mm) was used, and the temperature was set at 45 ℃. An ICDS-40A electrodialysis suppressor was used, and the current was set at 150 mA. The linear ranges of the eight organic acids and anions were 0.1-10.0 mg/L; their correlation coefficients (*r*) were ≥0.9993. The limits of detection (LODs) for the eight organic acids and anions were 0.0017-0.0150 mg/L based on a signal-to-noise ratio of 3, and the limits of quantification (LOQs) were 0.0057-0.0500 mg/L based on a signal-to-noise ratio of 10. The intra-day precisions were 5.06%-6.33% (*n*=5), and the inter-day precisions were 5.37%-7.50% (*n*=5). This method was used to detect organic acids and anions in the exhaled breath of five healthy subjects. The contents of organic acids and anions in the exhaled breath were calculated. The content of lactic acid was relatively high, at 1.13-42.3 ng/L, and the contents of other seven organic acids and anions were 0.18-11.0 ng/L. During a 10 km-long run, the majority of organic acids and anions in the exhaled breath of five subjects first increased and then decreased. However, due to abnormal metabolism, the content changes of lactic acid, acetic acid, pyruvic acid and chloride in one subject were obviously different from others during exercise, showing a continuous rise. This method has the advantages of involving a simple sampling process and exhibiting good precision, few side effects, and no obvious discomfort or risk to the subjects. This study provides experimental ideas and a theoretical basis for future research on human metabolites.

基于呼出气冷凝液(exhaled breath condensate, EBC)的分析是一种对人体气道内液体进行组分分析的无创方法^[1,2]^。EBC绝大部分由水组成,此外还有一小部分为人体呼出气中携带的微小肺泡内衬液滴,因此EBC中包含一些标志性分子,这些分子可以作为指示肺部疾病的生物标志物^[3,4]^。已有众多研究表明,EBC中某些成分的显著异常可反映哮喘、慢性阻塞性肺疾病、急性呼吸窘迫综合征、肺间质疾病等肺内及气道内氧化应激、炎症状态的变化及程度^[5,6]^。传统的肺部生物标记物收集方法(如肺泡灌洗、痰诱导等)大多是侵入性或半侵入性的,相比之下,呼出气冷凝液的收集与分析是一种非侵入性方法,受试者不会产生任何明显不适或风险,且操作简单、可重复。因此,呼出气冷凝液分析在医学诊断和治疗上具有广阔的发展前景。

由于人体呼出气中含有大量水蒸气,收集的EBC中待测目标物的浓度一般较低,因此需要更为精确的分析手段来测定EBC中各类目标物的浓度。已有研究采用生物传感器法、离子色谱法、气相色谱-质谱法、高效液相色谱-质谱法等手段对EBC进行分析^[7⇓⇓⇓⇓⇓⇓⇓-15]^。其中,离子色谱法是分析离子态化合物的有效手段之一,具有准确度高、灵敏度好、操作简便等特点,对EBC样品不需要复杂样品前处理,可用于多种阴离子和小分子有机酸的同时检测。在EBC中存在的多种物质中,乳酸已被证明是慢性阻塞性肺疾病和其他肺部疾病的潜在指标^[7]^。丙酮酸是一种*α*-酮酸,在生物体的代谢中起着举足轻重的作用^[16]^。Sauvain等^[15]^通过离子色谱检测和基于主成分分析的统计处理,证明暴露于石英会导致EBC样本中阴离子浓度的改变,并预测分析EBC样本中的阴离子含量可用于评估暴露于颗粒物(PM)引起的代谢反应。因此,测定EBC中的多种阴离子和小分子有机酸可为人体代谢研究提供基础数据,具有科研应用价值。

EBC中的待测目标物浓度受水蒸气稀释的影响较大,因此为保证分析结果的可靠性,需要建立一套标准的EBC收集方法。本研究设计并定制了一种可监测呼气流量、气体收集体积及冷却温度的EBC收集设备,对EBC收集的最佳采集冷阱温度和采集流量进行探究,并建立了基于该设备的EBC收集标准流程,采用离子色谱配合标准曲线法对受试者呼出气中8种阴离子及有机酸进行检测。

## 1 实验部分

### 1.1 仪器与试剂

HIC-ESP离子色谱仪、ICDS-40A电渗析抑制器、IC-SA3 (250 mm×4.0 mm)阴离子色谱柱(日本Shimadzu公司); Milli-Q超纯水机(美国Millipore公司)。碳酸钠、碳酸氢钠、乳酸、甲酸钾、乙酸钠、丙酮酸钠、氯化钠、亚硝酸钾、硝酸钾、硫酸钾均为分析纯(上海阿拉丁生化科技股份有限公司)。

人体呼出气冷凝液均于浙江大学医学院附属第二医院校医院院区收集,所有参与者均签署了书面知情同意书,并通过了浙江大学医学院附属第二医院伦理审查委员会的批准((2023)伦审研第(0557)号)。

### 1.2 实验装置

呼出气冷凝液采集装置为实验室定制,内部结构详见图1。该冷凝收集装置包括吹气口、与吹气口相连的单向阀和流量计、置于半导体冷凝装置中的冷阱,冷阱内设有一次性冷凝收集管。采集过程中,可在外部控制面板上监测实时呼气流量、冷阱冷凝温度与气体体积。

**图 1:**
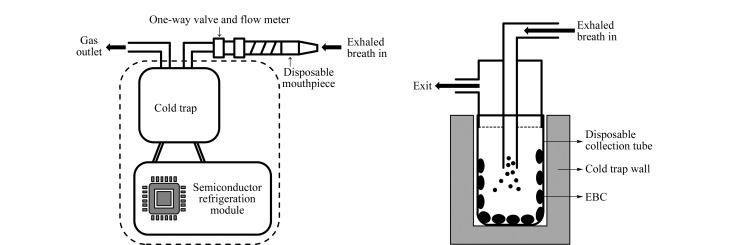
呼出气冷凝液采集装置示意图

### 1.3 色谱条件

分析柱:IC-SA3 (250 mm×4.0 mm);抑制器:ICDS-40A电渗析抑制器,抑制器电流:150 mA;流动相:1.5 mmol/L碳酸钠和3 mmol/L碳酸氢钠混合溶液;泵流速:0.8 mL/min;柱温:45 ℃;进样体积:100 μL。

### 1.4 样品的采集与分析

用定制的收集装置对受试者的呼出气冷凝液进行采集。采集前在仪器控制面板上设置采集冷阱温度-15 ℃及采样体积15 L/min,待冷阱温度冷却到设定值且保持平衡时开始采样。受试者用纯净水漱口再进行采样,采样过程中受试者深吸气至肺部充满气体后,通过吹气口均匀呼出,采集到设定的体积150 L(*V*)时仪器将发出提示声,立刻结束采集并记录呼气时间,根据呼气时间与采样体积计算平均采集流量。采集结束后,取出一次性冷凝收集管,测定并记录呼出气冷凝液的体积(*V*_1_, μL),密封后置于-20 ℃冰箱中避光保存。

收集的呼出气冷凝液用微孔滤膜过滤后,在1.3节的色谱条件下进行分析。扣除去离子水背景值后,根据标准曲线得到呼出气冷凝液中离子的质量浓度(*C*_1_,mg/L)。根据公式(1)计算得到人体呼出气中离子的质量浓度(*C*,ng/L)。


(1) *C=C*_1_*V*_1_*/V*

## 2 结果与讨论

### 2.1 采集冷阱温度和采集流量的优化

在健康受试者稳定状态下,保持呼气流量恒定,采集冷阱温度分别为-9、-12、-15、-18、-20 ℃,进行呼出气冷凝液的采集与分析,每个样品平行测定3次。利用公式(1)计算得到呼出气中有机酸和阴离子的含量(见图2)。在呼气过程中,气道黏膜液以微型液滴的形态与挥发性物质一同被呼出。随着采集冷阱温度的降低,小液滴逐渐附着于收集管壁,呼出气中有机酸和阴离子的采集效率呈上升趋势,当温度降至-15 ℃时基本冷凝完全,采集效率趋于稳定。因此,确定冷阱中最佳冷凝温度为-15 ℃。

**图 2:**
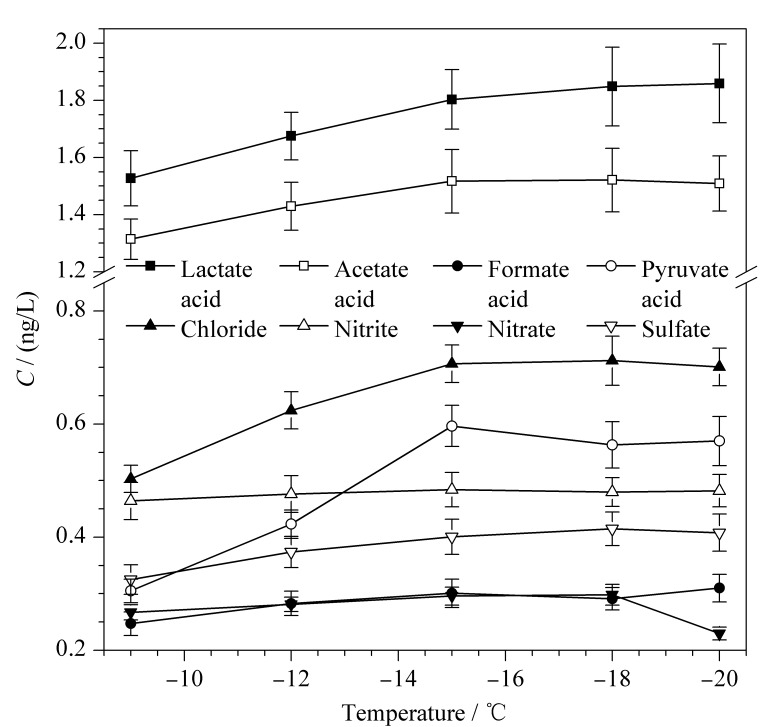
不同采集冷阱温度对呼出气中有机酸和阴离子含量的影响(*n*=3)

在健康受试者稳定状态下,保持采集冷阱温度为-15 ℃,呼气流量分别为5、10、15、20、25 L/min条件下进行呼出气冷凝液的采集与分析,每个样品平行测定3次。利用公式(1)计算得到呼出气中有机酸和阴离子的含量(见图3)。随着采集流量的增加,呼出气中有机酸和阴离子的采集效率先升高后降低。采集流量过高会导致呼出气成分来不及冷凝就被排出,流量过低会导致有部分液滴冷凝于橡胶管道中无法收集。因此,确定最佳呼气流量为15 L/min。

**图 3:**
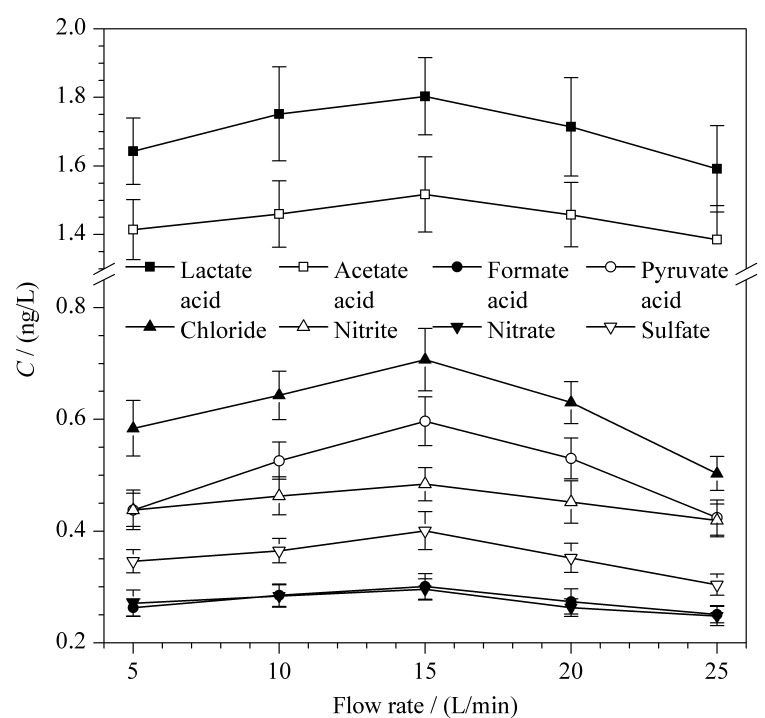
不同采集流量对呼出气中有机酸和阴离子含量的影响(*n*=3)

### 2.2 标准曲线及检出限

配制0.1、0.2、0.5、1.0、2.0、5.0、10.0 mg/L的8种目标物系列标准溶液。用微孔滤膜过滤后,在1.3节色谱条件下进行分析,标准溶液的色谱图见图4。扣除去离子水背景值后以峰面积*y*为纵坐标,质量浓度*x*为横坐标,拟合得到线性方程。8种有机酸和阴离子的线性范围均为0.1~10.0 mg/L,相关系数(*r*)均≥0.9993(见表1)。以3倍和10倍信噪比(*S/N*)确定呼出气冷凝液中有机酸和阴离子的检出限(LOD)与定量限(LOQ)。在进样量为100 μL时,8种有机酸和阴离子的LOD和LOQ分别为0.0017~0.0150和0.0057~0.0500 mg/L。

**图 4:**
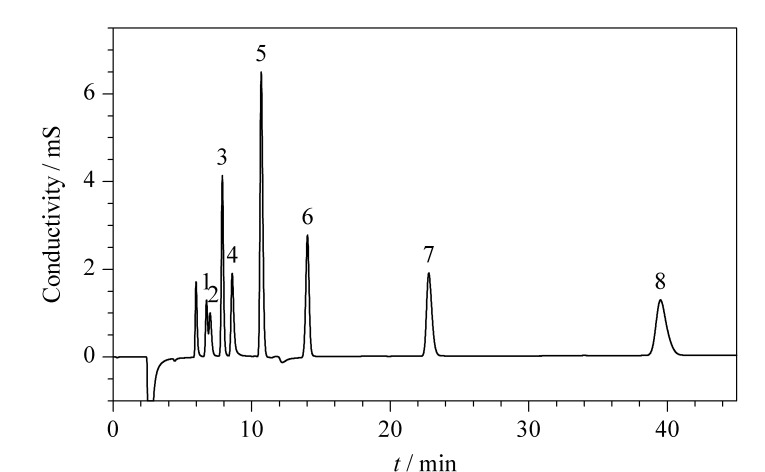
8种有机酸和阴离子的混合标准溶液色谱图

**表 1:** 8种有机酸和阴离子的线性方程、线性范围、相关系数、检出限和定量限

Analyte	Linear equation	Linear range/(mg/L)	r	LOD/(mg/L)	LOQ/(mg/L)
Lactic acid	y=13334.77x+301.17	0.1-10.0	0.9999	0.0098	0.0327
Acetic acid	y=12876.38x-21.85	0.1-10.0	0.9997	0.0150	0.0500
Formic acid	y=43544.47x+83.61	0.1-10.0	0.9993	0.0032	0.0107
Pyruvic acid	y=29861.26x-1957.85	0.1-10.0	0.9995	0.0087	0.0290
Chloride	y=90379.37x-615.57	0.1-10.0	0.9998	0.0017	0.0057
Nitrite	y=49210.76x-1491.45	0.1-10.0	0.9997	0.0059	0.0197
Nitrate	y=52598.70x-625.76	0.1-10.0	0.9994	0.0070	0.0233
Sulfate	y=67371.49x+266.48	0.1-10.0	0.9999	0.0082	0.0273

*y*: peak area; *x*: mass concentration, mg/L.

### 2.3 精密度

在健康受试者稳定状态下,一天内在相同状态下每间隔30 min采集一次呼出气冷凝液后进行分析,每个样品平行测定5次,得到日内精密度,为5.06%~6.33%;一周内每天在受试者相同状态时采集呼出气冷凝液进行分析,每个样品平行测定5次,得到日间精密度,为5.37%~7.50%。

### 2.4 实际样品分析

在5位健康受试者A~E稳定状态下,进行呼出气的采集与分析,每个样品平行测定3次。其中A、B、C为成年男性,D、E为成年女性。利用公式(1)计算得到呼出气中有机酸和阴离子的含量见表2。5位健康受试者呼出气中8种有机酸和阴离子的含量范围为0.18~42.3 ng/L。其中,乳酸含量相对较高,为1.13~42.3 ng/L,其他7种有机酸和阴离子的含量为0.18~11.0 ng/L。一个实际样品的色谱图见图5。

**表 2:** 呼出气中8种有机酸和阴离子的含量(*n*=3)

Analyte	A			B			C			D			E	
	C/(ng/L)	RSD/%		C/(ng/L)	RSD/%		C/(ng/L)	RSD/%		C/(ng/L)	RSD/%		C/(ng/L)	RSD/%
Lactic acid	14.5	4.24		21.6	6.36		42.3	6.78		1.13	4.57		1.80	7.45
Acetic acid	11.0	6.18		3.97	6.98		3.49	8.23		0.95	7.23		1.52	5.75
Formic acid	3.23	5.76		0.70	7.23		0.87	8.47		0.18	5.86		0.30	6.45
Pyruvic acid	0.75	6.82		1.00	6.25		1.21	4.86		0.58	5.67		0.60	6.17
Chloride	6.26	5.86		2.46	4.87		1.11	5.45		0.74	8.33		0.71	5.05
Nitrite	0.72	6.97		0.66	4.68		0.69	6.03		0.47	8.35		0.48	6.64
Nitrate	2.75	4.87		1.68	5.16		2.26	7.15		0.37	4.72		0.30	5.84
Sulfate	2.38	8.33		1.45	5.73		2.23	6.45		0.28	4.97		0.40	5.75

*C*: mass concentration of organic acids and anions in human exhaled breath.

**图 5:**
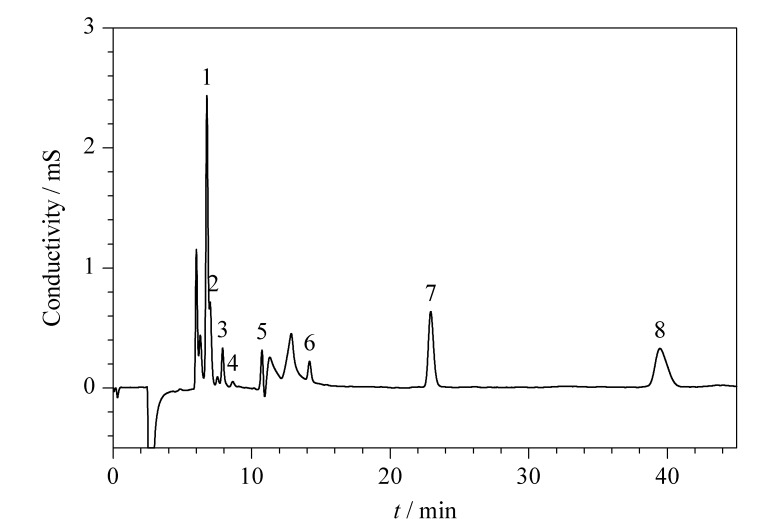
实际样品的色谱图

### 2.5 实际运动过程的应用

如图6所示,在5位健康受试者跑步前以及跑步1、3、5、10 km后,在最佳条件下进行呼出气的采集与分析,每个样品平行测定3次,记录运动过程中各离子的浓度变化趋势。可以看出,在10 km的长跑运动过程中,各受试者呼出气中的有机酸和阴离子含量总体变化趋势为先增加后减少,这可能是由于在剧烈运动时,人体内的代谢增加,并且主要的代谢方式为无氧呼吸,人体内的有机酸生成增多,汗液排出体外,同时随着人体呼吸变得急促,气道振动幅度增大,呼气微型液滴的含量增加,呼出气中有机酸与阴离子含量升高。在长时间的跑步后,5位受试者均达到极限状态,最后3~5 km的运动均转变成极慢速度的低强度慢跑,无氧呼吸转变成有氧呼吸,该过程中体内积累的代谢产物逐渐被机体清除,汗液不再排出,呼吸趋于平稳,呼出气中有机酸和阴离子含量降低。其中较为特别的是受试者B,其运动过程中乳酸、乙酸、丙酮酸和氯离子的含量变化明显与他人不同,表现为持续上升。经调查得知,受试者B在参与实验的时间段内服用了泰尔丝的异维A酸,该药品会对人体代谢造成一定影响,因此呈现出特殊结果。日后将通过进一步的研究证实两者的联系。

**图 6:**
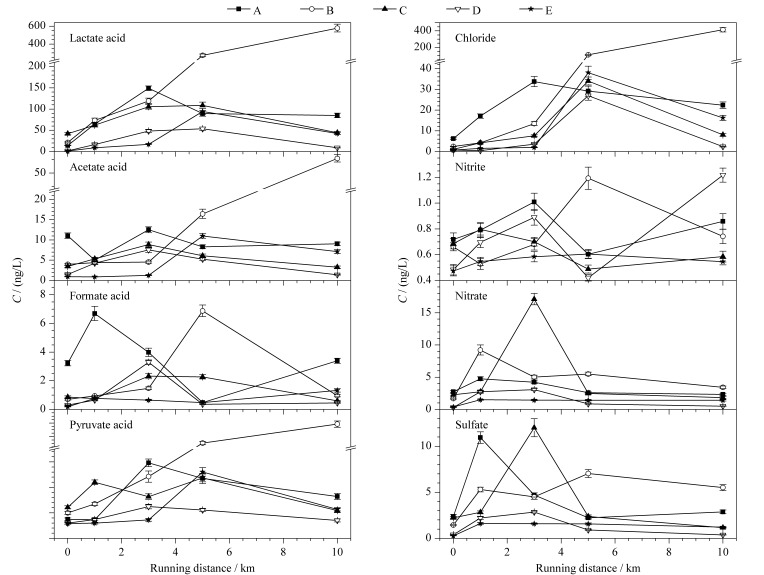
5名健康受试者运动过程中8种有机酸和阴离子的含量变化趋势(*n*=3)

## 3 结论

建立了一种非侵入式冷凝收集测定人体呼出气中有机酸和阴离子含量的分析方法。通过搭建自制呼出气冷凝设备对人体呼出气进行冷凝收集,将收集的EBC通过离子色谱进行8种有机酸和阴离子含量的检测。该方法采样过程简单、可重复,并且没有副作用,受试者不会产生明显不适或风险,可为日后人体代谢物的新型检测技术提供理论依据。
